# Draft Genome Sequences of Nine Stenotrophomonas maltophilia Isolates from a Freshwater Catchment Area in Hong Kong

**DOI:** 10.1128/mra.00238-22

**Published:** 2022-06-23

**Authors:** A. C. Y. Cheung, G. K. K. Lai, S. D. J. Griffin, F. C. C. Leung

**Affiliations:** a Shuyuan Molecular Biology Laboratory, The Independent Schools Foundation Academy, Hong Kong SAR, China; University of Delaware

## Abstract

Stenotrophomonas maltophilia is a widely distributed, Gram-negative bacillus that is increasingly identified as a multidrug-resistant opportunistic pathogen of concern. Here, we report the draft genome sequences of nine strains that were isolated from a freshwater catchment area in Hong Kong, corresponding to four different monophyletic lineages within the species.

## ANNOUNCEMENT

Stenotrophomonas maltophilia is a widely distributed, nonfermentative, Gram-negative bacillus that forms a complex of 23 monophyletic lineages, namely, Sm1 to Sm18 (including Sm4a and Sm4b) and Sgn1 to Sgn4 (1). An environmental species found in water (2) and soil (3) and frequently plant associated (4–6), it is considered a low-virulence pathogen (7). Nevertheless, it is responsible for some serious infections in hospitals, for example, among intensive care unit (ICU) patients (8, 9) and burn patients (10), and is the third most common source of secondary infection following severe and critical cases of coronavirus disease 2019 (COVID-19) (11). Of particular concern clinically is its multidrug resistance, including intrinsic resistance to carbapenems via an L1 and/or L2 metallo-β-lactamase (12–14). Contrasting with its pathogenicity in humans, S. maltophilia can promote plant growth (15) and has been proposed as an agricultural probiotic (16). Its resilience in challenging environments and its ability to degrade a wide range of substrates may also support a role in bioremediation (5, 17, 18). Gröschel et al. noted that, while its lineages are globally represented, they are not equal in their association with humans; for example, the authors found that Sm6 is most common among hospitalized patients, while strains within Sgn1 and Sgn2 appear entirely environmental (1).

Nine S. maltophilia strains were isolated during a survey of 10 sites within the catchment area of a freshwater stream in Telegraph Bay, Hong Kong. Aliquots (100 μl) of water samples collected at each site were initially spread on Luria agar containing ampicillin (100 μg/mL) and incubated at 27°C for 48 h. Resultant colonies were transferred to Luria agar containing amoxicillin-clavulanate (Augmentin) (100 μg/mL). Colonies resistant to both ampicillin and Augmentin were tested for resistance to cefepime (30 μg) and ertapenem (10 μg) (discs from Liofilchem). Colonies showing unrestricted growth in the presence of all of the β-lactam antibiotics tested were subsequently passaged eight times on standard Luria agar (19). Single colonies were then spread on Luria agar and incubated for 48 h before harvesting for DNA extraction (Qiagen DNeasy PowerSoil Pro kit). Paired-end short-read sequencing libraries were prepared using the NexteraXT DNA library preparation kit and sequenced via the Illumina MiSeq platform using v3 chemistry (2 × 300 bp). Adapter sequences were removed using Trimmomatic v0.32 (20), and reads were quality filtered and trimmed before assembly with Newbler v2.7 (Roche Diagnostics). Default parameters were used for all software unless otherwise specified. Draft sequences were submitted to NCBI PGAP v5.0 (21) and PATRIC (22) for annotation. Sequencing data and analysis results for all nine isolates are summarized in Table 1.

**TABLE 1:** Sequencing data and genomic analysis results

Strain	GenBank accession no.	SRA accession no.	BioSample accession no.	Estimated genome size (Mbp)^*a*^	G+C content (%)	No. of contigs	*N*_50_ (bp)	Avg read length (bp)	Avg read coverage (×)	No. of sequencing reads	No. of protein-encoding genes	No. of rRNA genes (5S + 16S + 23S)^*b*^	No. of tRNA genes	No. of pseudogenes	Lineage^*c*^
ACYCa.1J	JAIOAN000000000	SRR15841080	SAMN21163372	5.4	66.47	89	112,705	259	44	916,233	4,169	4 + 1 + 1	68	42	Sm6
ACYCb.1K	JAIOAM000000000	SRR15841079	SAMN21163373	5.1	66.55	49	152,546	267	41	794,343	3,908	2 + 1 + 1	69	30	Sm5
ACYCa.2H	JAIOAL000000000	SRR15841078	SAMN21163374	5.3	66.55	119	126,128	259	62	1,279,259	4,097	2 + 1 + 1	67	32	Sm6
ACYCc.3B	JAIOAK000000000	SRR15841077	SAMN21163375	5.2	66.82	80	102,700	271	30	589,756	4,046	2 + 1 + 1	68	40	Sm4a
ACYCa.6E	JAIOAJ000000000	SRR15841076	SAMN21163376	5.5	66.29	117	96,532	273	38	782,889	4,238	2 + 1 + 1	67	46	Sm6
ACYCb.6H	JAIOAI000000000	SRR15841075	SAMN21163377	5.0	66.52	48	186,150	261	57	1,096,649	3,918	4 + 1 + 1	67	34	Sm5
ACYCe.8N	JAIOAH000000000	SRR15841074	SAMN21163378	5.5	66.37	107	122,820	262	51	1,075,603	4,170	2 + 1 + 1	67	34	Sm6
ACYCd.9D	JAIOAG000000000	SRR15841073	SAMN21163379	5.4	66.55	70	205,490	258	47	996,651	3,916	2 + 1 + 1	67	41	Sm3
ACYCb.10K	JAIOAF000000000	SRR15841072	SAMN21163380	5.4	66.10	55	223,385	262	71	1,466,929	4,189	2 + 1 + 1	68	44	Sm5

a By Newbler v2.7.

b Found complete by NCBI PGAP.

c Determined by MinHash genomic distances (23) from representative strains (1).

Using MinHash genomic distances (23) from representative strains characterized by Gröschel et al. (1), the nine strains were classified into lineages Sm3, Sm4a, Sm5 (3 isolates), and Sm6 (4 isolates). All isolates carry the L1 metallo-β-lactamase, as well as *sul4* (24). ACYCe.8N also carries *sul2*, *katG* (25), and *catB11* (26–28).

### Data availability.

The GenBank, Sequence Read Archive (SRA), and BioSample accession numbers of all nine isolates are listed in Table 1 and may also be accessed under NCBI BioProject accession number PRJNA759338.
